# Emotions and arithmetic in children

**DOI:** 10.1038/s41598-022-24995-9

**Published:** 2022-12-01

**Authors:** Patrick Lemaire

**Affiliations:** Aix-Marseille Univ, CNRS, LPC, Marseille, France

**Keywords:** Psychology, Human behaviour

## Abstract

How do negative emotions influence arithmetic performance and how such influence changes with age during childhood? To address these issues, I used a within-trial emotion induction procedure while children solve arithmetic problems. More specifically, 8–15 year-old participants (*N* = 207) solved arithmetic problems (8 + 4 = 13. True? False?) that were displayed superimposed on emotionally negative or neutral pictures. The main results showed (a) poorer performance in emotionally negative conditions in all age groups, (b) larger deleterious effects of negative emotions on harder problems, (c) decreased effects of emotions as children grow older, and (d) sequential carry-over effects of emotions in all age groups such that larger decreased performance under emotion condition relative to neutral condition occurred on current trials immediately preceded by emotional trials. These findings have important implications for furthering our understanding of how emotions influence arithmetic performance in children and how this influence changes during childhood.

## Introduction

A number of studies found that adults’ cognitive performance is influenced by emotions^1–3^. Thus, in a variety of domains, performance can increase or decrease under emotion conditions when participants accomplish both domain-general (e.g., attention, memory, reasoning, decision making) and domain-specific (e.g., arithmetic, time perception, language, music) tasks. Similarly, emotions influence children’s cognitive performance in a wide variety of domains, including memory, attention, executive control, decision making, or reasoning and problem solving^4–9^. Surprisingly, despite numerous mentions of the important role of emotions in mathematics^10–13^, only a few studies examined how emotions influence arithmetic performance in children. The few existing studies have yielded inconsistent results. As a consequence, unclear is how emotions influence children’s arithmetic performance, and how effects of emotions change with age. The present study investigated how negative emotions influence arithmetic performance in children aged 8–15.

A large number of studies on children’s arithmetic found that children’s performance is influenced by problem^14^, situation^15^, task^16^, and participants’^17^ characteristics^18–20^. Thus, for example, children obtain better performance on easier problems, when they are asked to perform a problem-verification task (e.g., saying whether 8 + 4 = 13 is true or false) than a production task (e.g., find the answer to 8 + 3), or when they do not have too strong speed or accuracy pressures. How emotions impact children’s arithmetic performance is less clear however, although previous findings showed that adults’ performance is influenced by negative emotions.

Previous studies on effects of emotions on arithmetic performance have examined how individual differences in mathematics anxiety correlate with arithmetic performance or have, though more rarely, manipulated participants’ emotional states and investigated how changes in emotional states influence individuals’ arithmetic performance. Both lines of research suggest that participants do not solve arithmetic problems the same way under negative and neutral emotional states. In adults, high-math anxious individuals tend to have poorer performance than lower math-anxious individuals^21,22^, and arithmetic performance decreases when individuals are tested under conditions of social pressures^23^, or after failing at an unrelated task accomplished immediately before an arithmetic problem-solving task^24–26^. Also, when adults are asked to verify one-digit addition problems (e.g., 8 + 4 = 13. True? False?) or to estimate the results of two-digit multiplication problems (e.g., which estimate is closer to the correct product of 42 × 57, 2000 or 3000?), they obtained poorer performance on problems displayed superimposed on emotionally negative than on emotionally neutral images^27–32^.

In children, like in adults, math anxiety correlates with math performance, as early as pre-elementary and elementary schools^33–38^. Also, like in adults, children’s math performance decreases under pressures^39^, and is better with increased knowledge in emotions^40^. Experimental emotional induction yielded inconclusive findings. In one study, 8–10 and 14.7–17.6 year-old participants were asked to close their eyes and think of the happiest moment in their life or of the last time they felt very happy. After 45 s, they were asked to open their eyes and to describe what they were thinking of. Following this, they were given 50 addition and subtraction problems and asked to solve as many problems as possible in five minutes. Both younger and older children solved more problems following positive emotion induction. However, lack of control for non-emotional factors makes it impossible to know whether the positive emotion group obtained slightly better performance than the no-treatment group because of positive emotions or because the imagery task triggered focused attention and/or increased non-specific arousal that enabled participants to solve more arithmetic problems. Two studies^41,42^, using the same affective priming paradigm found inconsistent results. One study^42^ found that 7–13 year-old children were faster at verifying arithmetic problems following oral presentation of emotionally positive words and slower after negative words, while another study^41^ reported no effects of emotions in children of the same ages.

In the present experiment, 8–15 year-old participants verified one-digit addition problems that were displayed superimposed on emotionally negative or neutral pictures. If emotions disturb children while they solve arithmetic problems, they should obtain poorer performance under negative emotion than under neutral emotion condition. This could occur if negative emotions capture children’s attentional resources that cannot be fully allocated to arithmetic problem solving. Moreover, if deleterious effects of emotions decrease with children’s age, we should observe decreasing differences in performance between negative and neutral emotion conditions as children grow older. Such age-related changes are expected given that children’s arithmetic proficiency^18^ and emotional regulation skills^43–45^ increase during childhood.

This study was not pre-registered. The data can be found at https://osf.io/5rn7m/.

## Results

We first examined how effects of emotions on arithmetic performance change with participants’ age. Then, we investigated sequential modulations of effects of emotions on performance. In both analyses, latencies larger than the mean of the participant’s mean + 2.5 *SDs* were removed (*mean*: 0.5%).

### Age-related changes in effects of emotion on arithmetic performance

#### Effects of emotion on true and false problems

Effects of emotion on arithmetic performance and age-related differences therein were analyzed via mixed-design ANOVAs on mean response times for correct responses and percentages of errors (see Table 1), involving 4 (Age: 8, 10, 12, and 15 year-old children) × 2 (Problem Type: False, True) × 2 (Emotion: Neutral, Negative), with age as the only between-participants factor. We also analyzed *z* scores to control for potentially artifactual interactions due to increased speed of processing with age. Analyses of *means* and *z* scores showed similar patterns for effects of negative emotions on performance and for the Age x Emotion interaction. Therefore, only analyses of means are reported here. Also, the same age-related differences in effects of negative emotions came out significant when they were analyzed on proportional increased latencies in emotion condition relative to neutral condition (i.e., for each participant and each type of problem, the dependent variable was [(mean response times in the emotion condition – mean response times in the neutral condition)/mean response times in the neutral condition]. Finally, preliminary analyses examined effects of emotions during the first versus second half of the experiment. However, above and beyond general effects of block (i.e., children were faster during the second block than during the first block), no interaction involving the block factor came out significant. Therefore, we report analyses collapsed over blocks.

**Table 1:** Mean solution times and percentages of errors for true and false problems under neutral and emotion conditions as a function of children’s age.

Age group × problems	Latencies (in ms)			% Errors		
	Neutral	Emotion	Differences	Neutral	Emotion	Differences
**8 year old**						
True problems	6698	8627	1930	8.5	8.8	0.3
False problems	7152	8558	1407	8.8	8.8	0.0
Means	6925	8593	1668	8.7	8.8	0.2
**10 year old**						
True problems	3646	4087	441	3.4	4.3	1.0
False problems	3918	4228	310	4.3	4.0	− 0.2
Means	3782	4158	376	3.8	4.2	0.4
**12 year old**						
True problems	3506	3827	321	5.9	6.4	0.5
False problems	3521	3729	209	5.8	5.4	− 0.4
Means	3513	3778	265	5.8	5.9	0.0
**15 year old**						
True problems	2831	3124	292	6.8	5.4	− 1.4
False problems	2915	3166	251	7.0	5.7	− 1.3
Means	2873	3145	271	6.9	5.5	− 1.3

**Figure 1:**
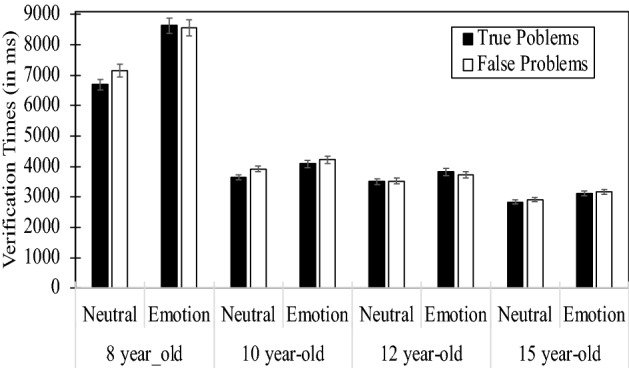
Age-related decrease in deleterious effects of emotion on children’s arithmetic performance.

Analyses of mean verification times showed main effects of age (*F*(3,203) = 65.105, *p* < 0.001, *ƞ*^2^_*p*_ = 0.490), problem type (*F*(1,203) = 4.912, *p* = 0.028, *ƞ*^2^_*p*_ = 0.024), and emotion (*F*(1,203) = 112.112, *p* < 0.001, *ƞ*^2^_*p*_ = 0.356). Children were faster as they grow older. Planned comparisons showed that 8 year olds (7559 ms) were slower than 10 year olds (3970 ms; *F*(1,203) = 104.59, *p* < 0.001, *ƞ*^2^_*p*_ = 0.340), who were as fast as 12 year olds (3646 ms; *F* < 1.0). Finally, 15 year olds (3009 ms) were marginally faster than 12 year olds (*F*(1,203) = 2.992, *p* = 0.085, *ƞ*^2^_*p*_ = 0.014). Moreover, participants were 105 ms faster on false problems than on true problems (4543 ms vs. 4648 ms), and 645 ms slower under emotion than under neutral condition (4918 ms vs. 4273 ms). Also, the significant Problem Type × Emotion interaction (*F*(1,203) = 8.551, *p* = 0.004, *ƞ*^2^_*p*_ = 0.040) revealed that emotions led participants to slow down more while verifying true problems (+ 746 ms; *F*(1,203) = 105.49, *p* < 0.001, *ƞ*^2^_*p*_ = 0.342) than false problems (+ 544 ms; *F*(1,203) = 65.35, *p* < 0.001, *ƞ*^2^_*p*_ = 0.244). This Problem Type × Emotion interaction was found significant in all age groups (*F*s > 8.503, *p*s < 0.005). Finally, and most interesting, the significant Age × Emotion (*F*(3,203) = 30.380, *p* < 0.001, *ƞ*^2^_*p*_ = 0.309) showed that effects of emotions decreased as children grow older (see Fig. 1), although emotion effects were significant in all age groups (*F*s > 19.272, *p*s < 0.001).

More specifically, emotions led 8 year olds to slow down by 1668 ms, 10 year olds by 376 ms, 12 year olds by 265 ms, and 15 year olds by 272 ms. Note that emotions increased latencies in 12 and 15 year-old participants to the same extent (*F* < 1.0). Analyses of errors showed that participants’ age was the only significant effect (*F*(3,203) = 7.652, *p* < 0.001, *ƞ*^2^_*p*_ = 0.102). Larger error rates were made by the youngest group of 8 year-old children compared to 10 year-olds (*F*(1,203) = 22.734, *p* < 0.001, *ƞ*^2^_*p*_ = 101), and comparable error rates were found among the other three age groups (*F*s < 3.623).

#### Distributional analyses of emotion effects

We used the so-called Vincentization technique^46^ to characterize the dynamics of the emotion effects and to compare these dynamics across age groups. We analyzed distributions of the emotion effects (i.e., latencies for emotion trials—latencies for neutral trials) as a function of the overall distribution of latencies^47,48^. The latencies for correct responses were sorted in ascending order and binned in four classes of equal size (*N* = 24 observations maximum). The mean of each bin (henceforth referred to as quartiles) was computed separately for each participant and each emotion condition. Average distributions of latencies were obtained by computing the mean values of quartiles by emotion condition (neutral, emotion), and age group separately. Preliminary analyses revealed similar distributions of emotion effects for true and false problems. Therefore, we report analyses collapsed over problem type.

**Figure 2:**
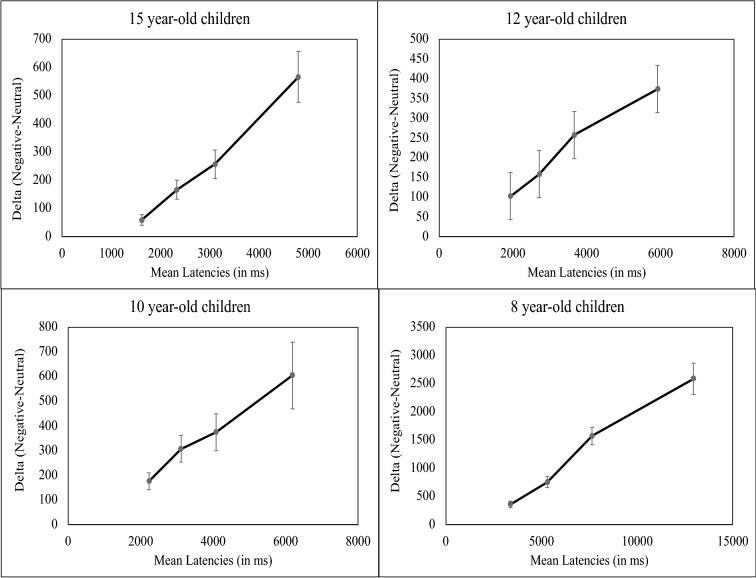
Distributions of emotion effects (delta plots) in each age. This plot shows how the size of emotion effects (differences in latencies between emotion and neutral conditions) varies as a function of the overall distribution of latencies for the 1st, 2nd, 3rd, and 4th quartiles.

Emotion effects were analyzed with an ANOVA with 4 (Age: 8, 10, 12, and 15 year-old children) × 4 (Quartile: 1st, 2nd, 3rd, and 4th), with age as the only between-participants factor. The main effects of age (*F*(3,609) = 28.925, *p* < 0.001, *ƞ*^2^_*p*_ = 0.299) and of quartile (*F*(3,609) = 54.192, *p* < 0.001, *ƞ*^2^_*p*_ = 0.211) were significant. The effects of quartiles were significant in all age groups (*F*s > 3.55), showing that the longer the latencies, the larger the emotion effects (linear trends, *Fs* > 4.451; see Fig. 2*).*

#### Mediation analyses

**Figure 3:**
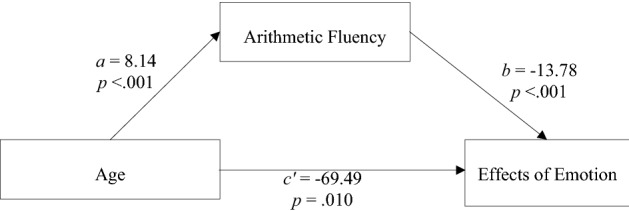
Simple mediation model for arithmetic fluency.

We tested whether arithmetic fluency mediated age-related changes in how emotions influence arithmetic performance. A simple mediation analysis on emotion effects (differences in latencies between emotion and neutral conditions) was carried out. Using the Medmod 1.1.0 module for JAMOVI (10,000 boostrapped resamples; Model 4^49^), we regressed emotion effects on age (coded in years) and entered arithmetic fluency as the mediator. As can be seen in Fig. 3, arithmetic fluency increased with increasing age (*a* = − 8.14), and the higher arithmetic fluency the smaller the effects of emotions (*b* = − 13.78). The confidence interval of the indirect effect through arithmetic fluency did not include zero (*ab* = − 112.30; CI 95% (− 169.00 to − 66.60). Arithmetic fluency was thus a significant mediator that accounted for 61.8% of the total age-related changes in effects of emotion on children’s arithmetic performance. Note however that age had a unique influence on emotional effects (*cʹ* = − 69.49, *p* = 0.010).

### Age-related changes in sequential modulations of effects of emotions

The goal of this series of analyses was to determine whether the emotion effects found on latencies on current trials were modulated by the type of immediately preceding trials (neutral vs. emotion) and to determine how such modulations change with age. Mean latencies on current trials (Table 2) were analyzed using 4 (Age: 8, 10, 12, and 15 year-old children) × 2 (Previous Trials: neutral, emotion) × 2 (Current Trials: neutral, emotion) ANOVAs, with age as the only between-participants factors.

**Table 2:** Mean solution times (in ms) and percentages of errors for current neutral or emotion trials following neutral or emotion trials.

Age × previous trials	Latencies (in ms)			% Errors		
	Current trials					
	Neutral	Emotion	Differences	Neutral	Emotion	Differences
**8 year old**						
Neutral	6915	8130	1215	9.2	8.5	0.7
Emotion	6670	8244	1574	8.4	9.0	− 0.7
**10 year old**						
Neutral	3724	4109	386	3.9	4.2	0.3
Emotion	3820	4250	430	3.8	4.1	0.3
**12 year old**						
Neutral	3507	3584	77	6.1	5.9	0.2
Emotion	3505	3933	428	5.5	5.9	0.3
**15 year old**						
Neutral	2815	3046	231	7.1	6.4	0.7
Emotion	2904	3267	363	6.6	4.4	2.2

The effects of age (*F*(3,203) = 64.399, *p* < 0.001, *ƞ*^2^_*p*_ = 0.869), previous trials (*F*(1,203) = 6.050, *p* < 0.015, *ƞ*^2^_*p*_ = 0.029), current trials (*F*(1,203) = 132.442, *p* < 0.001, *ƞ*^2^_*p*_ = 0.395), and Age × Previous Trials (*F*(3,203) = 27.075, *p* = 0.286, *ƞ*^2^_*p*_ = 0.002) were significant. Most importantly, the Previous Trials × Current Trials interaction (*F*(3,203) = 9.733, *p* = 0.002, *ƞ*^2^_*p*_ = 0.046) was significant. The Grade × Previous Trials × Current Trials interaction (*F* < 1.5) was not significant. In each age group, emotion effects on current trials were larger after emotion trials than after neutral trials. Emotion effects went from 1574 ms after emotion trials down to 1215 ms after neutral trials in 8 year-old children, from 430 to 386 ms in 10 year-old children, from 428 to 77 ms in 12 year-old children, and from 363 to 231 ms in 15 year-old children. All these emotion effects were significant (*t*s > 3.929, *p*s > 0.001), except after neutral trials in 12 year-old children (*t* < 1.190).

In sum, in addition to significant effects of emotion in all age groups, we found that deleterious effects of negative emotions on performance were larger on true than on false problems, increased with problems solved more slowly, and decreased with participants’ increasing age. Moreover, we found that effects of emotion were sequentially modulated by emotional valence of previous trials in all age groups, such that they were larger on current trials following emotion trials than after neutral trials.

## Discussion

To understand how emotions influence arithmetic during childhood, children aged 8–15 were asked to verify true/false, one-digit addition problems (i.e., 9 + 4 = 12. True? False?). Problems were displayed superimposed on emotionally neutral or negative images. The main findings showed that deleterious effects of negative emotions interacted with participants’ age and problem characteristics. More specifically, (a) children solved arithmetic problems more slowly in the emotion than in the neutral condition, especially harder problems, (b) deleterious effects of negative emotions on performance decreased with participants’ age, and (c) effects of negative emotions sequentially carried over across successive trials in all age groups such that they were larger on current trials immediately preceded by emotion trials. These findings have important implications for furthering our understanding of how emotions influence arithmetic performance, and how this influence changes with age during childhood.

We found that children of all age groups obtained poorer performance while solving arithmetic problems under negative emotions. Deleterious effects of negative emotions were larger for problems that participants needed more time to solve (e.g., true problems). This is the first direct evidence of effects of emotions on arithmetic in children. It is consistent with previous correlational findings showing that high-math anxious individuals have poorer performance than low-math individuals^21,22,33–35^. The present findings are also consistent with previous results showing deleterious effects of negative emotions while adults accomplish many cognitive tasks in general^1–3^ or solve arithmetic problems in particular^27,28,30–32,50,51^. This study generalizes deleterious effects of negative emotions to 8–15 year-old participants and specifies how these effects change with age.

The present poorer arithmetic performance under emotions in children can be explained like in adults^27,30^ by assuming that emotions capture participants’ attention and distract participants away from the target arithmetic problem-solving task. This attention hypothesis accounts for the present larger deleterious effects of negative emotions on true than on false problems. True problems require more cognitive resources than false problems to verify. With fewer available resources as a result of emotional processing, participants’ performance suffered more from negative emotions on true than on false problems in all age groups.

Interestingly, above and beyond general deleterious effects, emotions affected specific cognitive mechanisms as they influenced true problems more strongly than false problems. For both types of problems, participants needed to complete retrieval/calculation of correct answers before comparing them to the proposed answer, making a true/false decision, and responding. The difference between true and false problems concern comparison and decision-making mechanisms. Previous studies found that these mechanisms take longer for true than for false problems^52^. The present findings suggest that negative emotions increased latencies while executing these specific mechanisms, above and beyond influencing other mechanisms (e.g., encoding, retrieving correct answers in memory) required to verify arithmetic problems.

Although significant in all age groups, effects of emotions changed with children’s age. They decreased from age 8–12 and remained stable thereafter. Note that the effects of negative emotions were investigated here in a simple arithmetic problem solving task. It would be interesting to determine whether similar emotion effects and age-related changes in emotion effects occur when children are asked to solve more complex arithmetic problems. Given that these more complex problems are harder to solve, it is possible that different age-related changes in effects of emotions might be observed. For example, deleterious effects of negative emotions did not decrease with age after 12 year-old in the present study. It is possible that these effects of negative emotions would change in magnitude after age 12 for more complex arithmetic problems.

Our mediation analysis revealed that arithmetic fluency was a significant mediator of age-related changes in effects of emotion. This mediation analysis also showed that arithmetic fluency did not account for all the age-related variance in effects of emotions. This means that other factors contribute to how effects of emotions on arithmetic performance change with age during childhood. Future studies may investigate executive processing skills^53,54^ and emotion regulation^43–45^, known to undergo important growth in children. Altogether, increased arithmetic fluency, executive processing skills, and emotional regulation with age enable children to more and more efficiently block off or attenuate processing of task-irrelevant emotions and focus their attention to the target arithmetic problem-solving task as they grow older.

The final important findings in the present study concerns sequential carry-over effects of emotions*.* In all age groups, effects of emotions on current problems were larger after emotion trials than after neutral trials. This is similar to already found sequential difficulty effects in which participants obtain poorer performance on a given trial following a harder trial than after an easier trial. Such sequential difficulty effects have been found in several domains, including arithmetic^55–61^. They most likely result from accumulated interference effects of emotions across successive trials. This could happen if children did not disengage from emotions on previous trials when the next trials occur or if disengaging from negative emotions and solving arithmetic problems on previous trials deplete available resources. As a consequence, fewer resources would be available to solve problems on next trials.

The present findings have important implications for investigating the role of emotions on arithmetic performance. This role is currently studied via correlational studies (e.g., examining how individuals’ math anxiety correlate with arithmetic performance) or experimental studies (i.e., manipulating participants’ emotional states and determining how these manipulations change participants’ performance). Previous studies on math anxiety suggest that high-math anxious individuals obtain poorer arithmetic performance as a result of anxiety capturing processing resources while accomplishing arithmetic problem-solving tasks (see Dowker et al., 2016; Mamarella et al., 2019, for overviews). This is consistent with what happened in the present study in which children obtained poorer performance under negative emotion conditions than under neutral emotion conditions. This does not mean that the effects of math anxiety and of emotion induction are the same or result from the exact same mechanisms. Future studies will empirically determine the conditions under which both approaches yield similar effects (e.g., deleterious effects of math anxiety and manipulated negative emotions are larger on harder problems) and under which they result in different effects or similar effects of different magnitudes. Future studies will also further investigate how negative emotions influence arithmetic by combining both an experimental approach like here (i.e., using emotion induction procedures) and correlational approach (i.e., investigating effects of math anxiety). Such studies will further our understanding of the responsible mechanisms by which negative emotions influence arithmetic performance both in adults and in children.

Our account of effects of emotions on children’s arithmetic and age-related differences therein rely on implicitly assuming that children verified arithmetic problems with the same strategies under emotion and neutral conditions. However, previous research in children’s arithmetic found variability in strategy use when children solve arithmetic problems^62^. The possibility that children could solve arithmetic problems with different strategies under emotionally neutral and negative conditions could not be tested here, as arithmetic problem-verification tasks do not enable assessment of strategy use on a trial-by-trial basis with external behavioral evidence of strategies. Also, decreased deleterious effects of negative emotions on performance with increasing age during childhood may occur via age-related differences in how emotions influence which strategies children use and/or how children execute and select strategies on each trial. Negative emotions could lead younger children to use less efficient strategies more often than older children, to execute, and/or select them less efficiently on each problem. Future studies may test this possibility by examining whether effects of emotions on strategic aspects of arithmetic performance change with age during childhood.

## Methods

### Participants

We tested 207 8–15 year-old participants (see participants’ characteristics in Table 3). With no previous studies of negative emotions on children’s arithmetic, we determined sample size on the basis of previous studies on emotion and arithmetic in adults (e.g., Fabre & Lemaire, 2019; Lallement & Lemaire, 2021), where effect size of emotion on arithmetic performance ranged from 0.25 to 0.40, we used a *η*^2^*p* = 0.25 to determine the present sample size. With one between-participants factor (age) and two within-participants factors (problem type and emotion), our design could achieve 95% power with 76 participants. In order to exceed this criterion, we recruited 207 participants. Written informed consents were obtained from participants’ parents and oral consents were obtained from each participant. This research was approved by the National and Local Ethics Committees (Ref #: Comité Nationale pour la Recherche Impliquant des Personnes Humaines, CNRIPH 20.04.02.47414/2021-A01372-39) and was conducted in accordance of the Declaration of Helsinki.

**Table 3:** Participants’ characteristics.

Characteristics	8 y.o. (*N* = 49)	10 y.o. (*N* = 54)	12 y.o. (*N* = 52)	15 y.o. (*N* = 52)	*F*
*N* (females)	49 (22)	54 (19)	52 (24)	52 (27)	–
Mean age in years, months (SD)	8.4 (0.51)	11.3 (0.39)	12.5 (0.56)	15.6 (0.52)	1509.424**
Age range	8–10	10–11	11–14	14–16	–
Grade	Third	Fifth	Seventh	Tenth	–
TTR arithmetic fluency (SD)	50 (14.73)	81 (20.38)	89 (25.51)	109 (22.21)	66.085**

** *p* < 0.001.

### Stimuli

#### Arithmetic problems

Each child solved 96 problems. The basic set of problems included 12 individual addition problems presented in a standard form (i.e., *a* + *b*) with the operands *a* and *b* being one-digit numbers (e.g., 3 + 4; see Table S1 in Supplemental Material for the list of problems). Each individual problem was presented in *a* + *b* and *b* + *a* versions (e.g., 3 + 4 and 4 + 3). Each problem was presented with its correct sum (e.g., 3 + 8 = 11) and with an incorrect sum (e.g., 3 + 8 = 13). Proposed sums in false problems had a deviation of ± 1 or ± 2 units from correct sums. All true and false problems were presented once with a negative emotion picture (e.g., a car accident) and once with a neutral picture (e.g., a landscape). All in all, each participant solved 96 problems: 12 (individual problems) × 2 (versions: *a* + *b*, *b* + *a*) × 2 (true, false) × 2 (negative, neutral emotions). No tie problems (e.g., 3 + 3 = 6) were used; none of the operands were equal to 0, 1, 2, or 5; and none of the false answers were table-related products (e.g., 3 + 4 = 9). An additional set of 12 practice problems (similar to but different from experimental problems) was selected (see Table S2 in Supplemental Material for the list of practice problems).

#### Pictures

One hundred and eight pictures (96 for experimental trials and 12 for practice trials) were selected from the *Developmental Affective Photo System* (DAPS^64^). Half the pictures were emotionally negative (*mean* valence = 4.3; *SD* = 0.1 and *mean* arousal = 2.2; *SD* = 0.4), and half were emotionally neutral (*mean* valence = 2.7; *SD* = 0.3 and *mean* arousal = 3.8; *SD* = 0.3). Half the true and false problems were presented with emotionally negative pictures, and half with emotionally neutral pictures. In addition, arousal and valence were controlled across true and false problems (see Table 4).

**Table 4:** Mean valence and arousal in each category of problems (*SDs* in parentheses).

	True problems	False problems	*Fs*
Mean valence (neutral)	2.65 (0.29)	2.69 (0.32)	0.214
Mean valence (negative)	4.32 (0.13)	4.32 (0.18)	0.009
Mean arousal (neutral)	3.70 (0.31)	3.81 (0.34)	1.272
Mean arousal (negative)	2.16 (0.34)	2.25 (0.43)	0.593

In DAPS, valence ranges from 1 (very happy) to 5 (very unhappy), and arousal ranges from 1 (very excited) to 5 (very calm).

### Procedure

**Figure 4:**
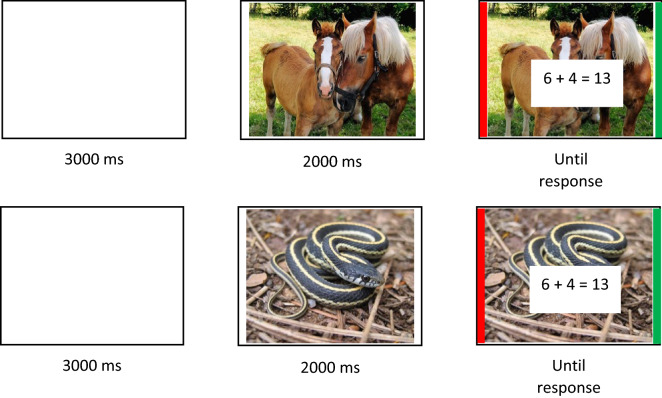
Illustration of procedure (images are from the OASIS database and were not used in this experiment as images used in this experiment were from DAPS).

The experiment was run on a Windows 10 Microsoft Surface Go2 Touch-Screen (10.5 inches), intel® Core™, M3-8100Y). Children were tested in groups of 10–20 participants in their classroom at school. Participants were told that they will see pleasant and unpleasant pictures and will complete an arithmetic problem-verification task. Each trial started with a 3000-ms blank screen (see illustration of a trial in Fig. 4). A picture was then displayed for 2000 ms. Then, addition problems together with a proposed sum appeared superimposed on emotionally neutral (e.g., landscape) or negative (e.g., a car accident) pictures until the participant’s response. Participants had to indicate if the proposed result was correct or not, as quickly and as accurately as possible. To do this, they had to press a delimited area (a 1.4-cm thick green stripe) on the right side of the Touch Screen if the proposed result was correct and a delimited area (a 1.4-cm thick red stripe) on the left side of the Touch Screen, if the proposed result was incorrect. Participants started with a practice session in which they verified 12 similar (but not the same) equations (6 true, 6 false, half with a neutral picture and half with a negative picture). Then, participants completed 96 trials, divided into two blocks of 48 trials each. Participants took a short break in-between the two blocks. The problem and pictures remained displayed on the screen until participant’ response. All problems were randomly presented to each participant.

At the end of the experiment, each participant saw 20 emotionally positive pictures (e.g., a smiling baby) in order to end the experiment in a positive mood. Each picture included a green or a red butterfly. On each picture, participants were asked to press on a green stripe (right side of the screen) if the butterfly on the picture was green or on a red stripe (left side of the screen) if the butterfly was green.

After this problem-verification task, participants’ arithmetic fluency was assessed via the *Arithmetic Tempo Test* (Tempo Test Rekenen^65^). This test includes four sets of 40 arithmetic problems each, with each set including only addition, subtraction, multiplication, or division problems. For each set, children had to provide the correct answer to as many problems as possible in 1-min. Finally, they had one minute to solve as many problems in a mixed set of addition, subtraction, multiplication, and division problems. The sum of correctly solved problems was calculated for each individual.

## Supplementary Information

Supplementary Information.

## Data Availability

The raw data of this study can be found at https://osf.io/5rn7m/.
