# Supplementary material for: Emotions and arithmetic in children
Source: Sci Rep. 2022 Dec 1;12:20702. doi: 10.1038/s41598-022-24995-9 (PMC9715942; doi:10.1038/s41598-022-24995-9)
Supplement: Supplementary file 1 — Supplementary Information. [file 41598_2022_24995_MOESM1_ESM.pdf]

## Supplementary Material

Table A1: Set of 12 individual problems. Each problem was seen by each individual, once in an *a*+*b* (e.g., 4+3) and once in a *b*+*a* (e.,g., 3+4) versions, and in the sound and silence conditions (96 problems/participant).

| Operands | True Answers | False Answers |
|---|---|---|
| 4+3 | 7 | 5 |
| 6+3 | 9 | 7 |
| 6+4 | 10 | 11 |
| 7+4 | 11 | 13 |
| 7+6 | 13 | 11 |
| 8+3 | 11 | 13 |
| 8+6 | 14 | 13 |
| 8+7 | 15 | 17 |
| 9+3 | 12 | 11 |
| 9+6 | 15 | 13 |
| 9+7 | 16 | 17 |
| 9+8 | 17 | 19 |

Table A2: Set of 12 problems used for baseline measures of latencies. Each problem was seen by each individual once in an *a*+*b* (e.g., 4+9=13) and once in a *b*+*a* (e.g., 9+4=13) versions in a silence condition.

| True Problems | | False Problems | |
|---|---|---|---|
| 3 + 5 = 8 | 4 + 9 = 13 | 3 + 5 = 9 | 4 + 9 = 11 |
| 3 + 7 = 10 | 5 + 8 = 13 | 3 + 7 = 12 | 5 + 8 = 12 |
| 5 + 7 = 12 | 5 + 9 = 14 | 5 + 7 = 11 | 5 + 9 = 16 |
